# Towards the just and sustainable use of antibiotics

**DOI:** 10.1186/s40545-016-0083-5

**Published:** 2016-10-07

**Authors:** Gemma L. Buckland Merrett, Gerald Bloom, Annie Wilkinson, Hayley MacGregor

**Affiliations:** 1 Health Action International, Overtoom 60 (2), 1054 HK Amsterdam, The Netherlands; 2 Institute of Development Studies, Library Road, Brighton, BN1 9RE UK

**Keywords:** Antibiotic/antimicrobial resistance, Equity, Justice, Sustainability, Systems, Pluralism

## Abstract

The emergence and spread of antibiotic resistant pathogens poses a big challenge to policy-makers, who need to oversee the transformation of health systems that evolved to provide easy access to these drugs into ones that encourage appropriate use of antimicrobials, whilst reducing the risk of resistance. This is a particular challenge for low and middle-income countries with pluralistic health systems where antibiotics are available in a number of different markets. This review paper considers access and use of antibiotics in these countries from a complex adaptive system perspective. It highlights the main areas of intervention that could provide the key to addressing the sustainable long term use and availability of antibiotics.

A focus on the synergies between interventions addressing access strategies, antibiotic quality, diagnostics for low-resource settings, measures to encourage just and sustainable decision making and help seeking optimal therapeutic and dosing strategies are key levers for the sustainable future of antibiotic use. Successful integration of such strategies will be dependent on effective governance mechanisms, effective partnerships and coalition building and accurate evaluation systems at national, regional and global levels.

## Background

The emergence and spread of bacteria resistant to existing antibiotics is of growing global concern [1]. It is widely recognised that low- and middle-income countries (LMICs), where the majority of the world’s population live, not only face particular challenges in addressing antibiotic resistance but also bear a disproportionate burden [2]. In these countries the spread of resistant bacteria is facilitated by poor hygiene, contaminated food, polluted water, overcrowding, and increased susceptibility to infection because of malnutrition, chronic illness and/or immunosuppression [3]. At the same time, factors such as the likely inappropriate use of antibiotics and availability of substandard antibiotics are rapidly driving resistance. In LMICs with weak health systems, the effect of antimicrobial resistance on health and economics is largely underestimated and incompletely understood. A common feature of these countries is the emergence of pluralistic health systems where government provision and health markets combine and where people obtain much of their antibiotics in unorganised markets with a wide variety of medicine providers [4]. A particular challenge in these health systems is the simultaneous existence of limited access to effective treatment of infections and high levels of antibiotic use. In this paper we develop an equity/social justice perspective which takes the conditions in low resource settings pluralistic health systems more fully into account than hitherto has been done. We review strategies and evidence for dealing with antibiotic resistance and consider how inequalities in health systems may influence their sustainability.

It is increasingly accepted that addressing antibiotic resistance requires a system perspective [5]. This is due to the myriad of interlinked technologies, networks, markets, regulations, perceptions, norms and infrastructures that influence antibiotic use. To be truly effective, efforts need to include strategies that cover pharmaceuticals, food and agriculture, human resources, financing, and information systems by linking science to practicality [3]. For an intervention to stand a good chance of success the relationships between diverse aspects and levels of the system need to be considered. The relevant system includes suppliers and users of antibiotic drugs and the local, national and global actors who influence them. Broadly conceived interventions are likely to be more robust. Elsewhere in health policy analysis there is increasing attention to the complex adaptive nature of health systems [6] and the importance of institutional arrangements and history which create path dependency [7]. This makes some interventions more appropriate than others in different contexts. These perspectives imply that the systems approach relevant to antibiotic use goes beyond acknowledging the existence of a multiplicity of actors to include their perspectives, interests, and the multi-dimensional norms and institutions which have developed around drug use over significant periods of time and in the context of severe resource constraints and inequity. Connections between individual and collective action must also be made.

In many LMIC settings characterised by this complexity one can also find strong belief in the efficacy of antibiotics with access viewed almost as a citizen’s entitlement [8]. Ensuring universal appropriate access to antimicrobials is not only a critical part of realizing the right to health, it also raises a number of ethical challenges surrounding distributive justice, individual liberty and the responsibility for the wellbeing of future generations. Designing interventions which are in line with/capture different notions of entitlement and justice, especially at local and national levels, will be central to sustainable, coherent and effective action against the development and spread of resistance. The need for both effective strategies to ensure improved and equitable access to antibiotics and strategies to ensure that providers and users are influenced to use them appropriately is at the cornerstone of tackling antibiotic resistance in LMIC contexts. Identifying the underlying conditions of antibiotic use and access presents key levers for balancing access and appropriate use at scale. This article presents an assimilation of the key areas for intervention and the challenges that need to be addressed in order to achieve just and sustainable use of antibiotics.

The objective of this article is to better understand how we can re-think the complex system of human antibiotic use in pluralistic health systems and measures to address the challenges, taking into account the conditions which influence sustainability in terms of access and long term efficacy. Although we recognise the importance of a ‘One Health’ approach to tackling the complexities of antibiotic resistance, this paper focuses on the human health perspective and the analysis is therefore limited accordingly.

## Methods

The data for this general review were identified by a search of PubMed (January 1966 to April 2016) as well as bibliographic references from relevant articles, including reviews on this subject and all selected studies. The inclusion search terms used were ‘antibiotic’ and ‘access’ or ‘excess’ or ‘rational use’ or ‘inappropriate use’. All relevant studies in the English-language literature that described access and appropriate use of antibiotics were assessed. Only studies with an explicit geographic focus on Low and Middle Income Countries or low resource locations were selected for use. This represented 30 % of articles retrieved. In addition, the focus of this review is human antibiotic use and therefore only those references describing human use were selected.

## Review

### Use of antibiotics in pluralistic health systems

The use of antibiotics in a pluralistic health system is driven by a number of social-technical dimensions, actors and factors influencing providers. The perceived value of antibiotics has diverged from their real value and thus created a system of use that is not always optimum. This is largely due to the way antibiotics are embedded in meanings, networks, markets and norms.

A common feature of pluralistic health systems is the variety of providers of health care and drugs with asymmetries in training, understanding, skills and varying relationships with formal regulatory systems. A number of factors, including established treatment practices and financial incentives, influence how these providers perform [9]. The porous boundaries between public, private, mission and NGO-sector providers means antibiotics can be accessed outside of regulatory frameworks and can be of differing quality. Other actors that can influence how antibiotics are used and accessed include those providing key information such as governments, mass media, NGOs, advocacy groups, advertising agencies. Patients are key actors in the health system, especially when antibiotics are available over-the-counter with the opportunity to self-medicate patients will be influenced by time, financial factors, perceived risk, and so forth [10].

Some pervasive beliefs and meanings have been attached to antibiotics which influence how they are used [11]. For example, the consistent promotion of antibiotics as part of public health programs and messaging from pharmaceutical companies has put emphasis on accessing antibiotics rather than on rational use. The ‘syndromic management’ approach that treats presumptively by trying to categorise diseases/conditions by symptoms in the absence of better diagnostics is also thought to be a major driver of resistance [12]. In some cases, recommendations of mass or presumptive treatment has cultivated practices where antibiotics are used indiscriminately and/or pre-emptively as opposed to a disease-specific manner. Their connotations of modernity and associations with Western medicine have also purveyed a desirable status for antibiotics [10].

### Interventions and the complex adaptive system

**Fig. 1:**
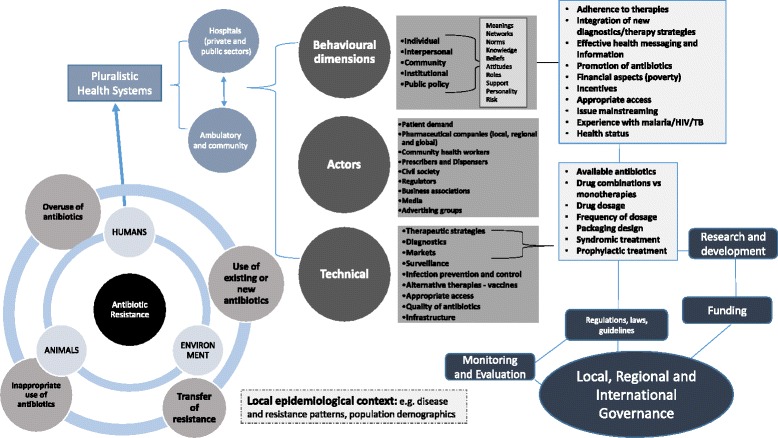
A complex system: human drivers of antibiotic resistance in pluralistic health systems

Policy makers are developing National Action Plans based on the WHO Global Action Plan approved at the 68th World Health Assembly. Whilst country-specific interventions will be needed there are a number of similarities when considering the key levers of intervention for pluralistic health systems. How countries provide access to efficacious antibiotics whilst ensuring rational use for future sustainability requires simultaneously targeting several key drivers and the underlying causations within a complex system. Figure 1. provides a system perspective of the manifold drivers of antibiotic resistance in community settings of pluralistic health systems and highlights the potential levers for intervention.

The following subsections outline some of the key themes that arise when considering possible interventions to tackle antibiotic resistance at the community level, along with the barriers and challenges that need to be addressed in order to develop sustainable interventions.

#### Access strategies

How should the flow of antibiotics be controlled in a system? Current thinking proposes interventions such as, restriction of formularies, exclusive vendor availability, requirement of preauthorisation of antibiotic use, and de-escalation of broad-spectrum coverage when a pathogen is identified [13, 14]. It will be especially important to ensure the perspective of the end user in considered, particularly the most under-served, within a system of controlled distribution and use of a new antibiotic. There is indeed a paucity of information regarding the extent of access to efficacious antibiotics in various localities. An understanding of the interplay of logistical, environmental, financial and social drivers behind current access patterns is a key component of developing more controlled access strategies. Effective collective action needs to be underpinned by a consensus on the need for action and a belief that measures taken are just [15]. In some cases, a policy tension might arise between saving lives with short-term mass campaigns that advocate antibiotics, and increased mortality as a result of increased antibiotic resistance. Learning from success stories in other countries will be an important part of developing effective and innovative interventions.

Central to the discourse on treatment options is the overall availability of antibiotics which determines the access that people have. Rises in the availability of low cost generic drugs have been shown to lead to increased consumption of antibiotics, with beneficial health outcomes [16]. However, it has also contributed to the emergence and spread of bacterial resistance to antibiotics. In some cases, it has encouraged the use of old drug products with very poor oral bioavailability, marketed with historical dosage regimens and extensively excreted in the environment. New business models are needed in human and veterinary health that make good quality and eco-friendly antibiotics available at an affordable price. Also new approaches are needed to encourage the development of new and innovative products that will be required to meet the therapeutic needs of the veterinary community while being consistent with public health concerns.

#### Quality

Beyond the harmful effects for patients, substandard medicines favour the emergence of bacterial resistance with a worldwide impact. Despite regulatory efforts, it is clear that substandard medications continue to be a major concern [17]. Substandard medicines from approved manufacturers still reach the market in relatively high volumes even when there are stringent quality assurance methods in place such as the WHO prequalification program. It is also difficult to pinpoint whether low quality manufacturing or poor storage conditions are responsible for the substandard quality of the medications. Several studies have found that location of purchase was an important indicator of quality as the failure rate for medications purchased from an unlicensed outlet was much higher than for those purchased from a licensed outlet [18, 19].

Ensuring access to antibiotics that are of an efficacious quality is of paramount importance when considering measures to foster just and sustainable access to antibiotics and how interventions to improve access will impact the quality of these medicines. Adequate regulatory capacity will need to be provided in all settings to ensure this. Equally important in the efforts to reduce the prevalence of substandard and counterfeit medications is the development of accurate yet low-cost testing mechanisms that can be easily applied in low-resource settings. An important distinction between issues of quality due to substandard storage or manufacturing and the issue of counterfeit medications is also essential to ensure the context of the problem is not conflated with discussions on intellectual property law [20].

#### Decision making and help seeking: changing patterns of use

In exploring strategies for improving the use of antibiotic drugs it is useful to analyse the health system as a knowledge economy, which makes the benefits of expert medical knowledge and specialised commodities, such as drugs, widely available [7]. An important characteristic of the health knowledge economy is the asymmetry in knowledge between experts and the people who rely on their advice.

Considering what determines human behaviour, and how we explain that behaviour are helpful questions to guide understanding what motivates people to modify their behaviour and to accept suggestions for modification. Behaviour change should be viewed from all aspects of antibiotic prescribing, dispensing, use, and handling and also understanding the causes that lead to unnecessary use of antibiotics. We identify five levels of factors that influence antibiotic use: 1) Individual—knowledge, attitudes, beliefs, and personality; 2) Interpersonal—social identity, support, roles; 3) Institutional—rules, guidelines, regulations, and informal structures; 4) Community—social networks, norms; and 5) Public policy—regulations and laws [21].

On the demand side, self-medication by consumers with antibiotics purchased without a prescription is common. Consumers have positive attitudes towards antibiotics, but paradoxically they have poor knowledge about these drugs and diseases [22]. The availability of antibiotics without prescription—an important enabling factor—mainly results from absence of prescription-only regulation, ineffective law enforcement, practices driven by poverty, culture, and norms [23]. Sub-optimum compliance in use, including taking leftover antibiotics from previous treatment courses and sharing unused drugs with other people, is common in both developed and developing countries [24]. Ideally, consumers should have access to accurate information on antibiotics and infectious diseases instead of access to antibiotics without prescription. When irrational use of antibiotics repeatedly happens among the public and health professionals, it becomes the norm. To break this pattern, antibiotic stewardship programmes should focus not only on appropriate use, but also on ensuring sustainability of behavioural change at all levels of the system and reorientation of social and institutional norms [5]. Solutions need to focus on multifaceted and multilevel interventions that define local barriers and beliefs, which can vary widely between cultures, countries, and regions. Education of all health-care workers, laboratory staff, veterinarians, and the public on appropriate antibiotic use and antibiotic resistance is essential, and educational strategies have recently been reviewed [25]. Although education alone might not be powerful enough as an intervention, it generates knowledge that is essential for health-care workers to understand and support the resistance control programmes. Education should be tailored and started early on to shape behaviour rather than having to change it. The potential benefits of using mobile health technologies (mHealth) in the transmission of health behaviour messaging, in addition to its use for tracking, reporting, messaging and the surveillance of resistance, needs to be explored further [26].

On the supply side, physicians are often role models for other health professionals and patients who learn how to use antibiotics from their prescriptions. Apart from medical training, physicians are influenced by their peers, and perceived demands of patients. Therefore, physicians might find it difficult to comply with treatment guidelines [11]. These barriers to compliance should be removed or minimised, and options for alternative actions for guideline compliance should be simultaneously provided. Examples of options for non-antibiotic treatment in viral or self-limiting infections are the prescription of herbal medicines, as opposed to antibiotics and use of a delayed prescription technique with explicit instructions for patients about when to use antibiotics [27]. To encourage guideline compliance, consequences of irrational use of antibiotics should be reframed to be relevant to the self-interest of prescribers and institutions. Motivational measures include pay-for-performance policy [27], the audit-feedback mechanism on antibiotic prescribing rates of individual prescribers [28], and public disclosure on antibiotic prescribing rates of each health-care facility or area [3]. Major challenges arise when antibiotic prescriptions are a source of revenue for individuals or institutions, either by a fee-for-service remuneration scheme [29] or drug-promotion incentives [30]. A recent Cochrane systematic review on the comparison of educational and persuasive versus restrictive methods for improving antibiotic prescribing demonstrated that on average, restrictive methods were three times more effective than persuasive interventions [31]. Prescribers and manufacturers also need to be aware that studies have shown a mismatch between antibiotic pack sizes and guideline recommendations for their duration is contributing to antibiotic resistance in the community. This opens up the potential need for more effectively designed packaging to combat resistance [32].

#### Therapeutic and dosing strategies

Antibiotic resistance presents a major scientific challenge; not only in developing potential new treatments and monitoring patterns of resistance but also in understanding the best protocols for treatments [33]. Even if new antibiotics are developed a re-evaluation of the best therapeutic models to employ in order to preserve and or enhance the antibacterial effects of available drugs will be necessary. Assumptions have been made on the best practices for prescribing and treating bacterial infections using antibiotics which are now beginning to be challenged [34]. The way therapies prescribed needs to be based on the most accurate data and scientific understanding in all population settings.

Therapeutic strategies are a much needed area of further research. In many cases, symptoms should guide the length of treatment, except for particular diseases where symptoms do not reflect the true pathogen load. In terms of treatment, emerging thoughts focus on the need to reassess the public health message surrounding the ideal duration of an antibiotic course; evidence shows that many infections clear with less than a typical course of antibiotics. Conversely, there are also arguments for using more aggressive doses to reduce the survival of resistant bacteria [35]. Promotion of sequential use, cycling strategies or mixing of different antibiotics have all demonstrated positive effects on the reduction of antimicrobial resistance [36]. There is also a need to look more thoroughly at the scientific and a societal perspective of drug combinations as opposed to monotherapy use to effectively combat the drug resistance phenotype - taking the lead from successful efforts seen with HIV and malaria therapies [37]. Several practicalities present challenges to implanting combination therapies, many of which have arisen in the case of malaria. These include the selection of drugs based on cost, ease of administration, acceptability, current levels of resistance, impact of combinations of drugs with mismatched half-lives, how the drugs are used (co-administration, co-formulation, duration, costs, understanding of regimes etc, cycling) and the economics of combination drug therapy [38].

As a first step, the global scientific community will need to strengthen its assessment of appropriate usage, defining parameters for deciding which antimicrobials are effective in which areas of the world and useful at various levels of health care systems. This is an evidence-based normative process, and is reflected in the development of previous effective antimicrobial programs. Furthermore, this is not a static effort, but needs to be continuously reviewed and updated based on dynamics of use and evidence of emerging resistance.

#### Diagnostics

Diagnostic tests play a major role in the detection of specific pathogens, discovery of new pathogens, determining appropriate therapy, monitoring response to therapy, assessing prognosis, and disease surveillance at the local, regional and national level [39]. Despite the increased use of rapid tests and the availability of molecular and proteomics-based tests, diagnostics are not being integrated into clinical care optimally [40]. Many patients with suspected infections receive empiric antimicrobial therapy rather than appropriate therapy dictated by the rapid identification of the infectious agent. The result is over use of a small inventory of effective antimicrobials. Similarly, the reliance on syndromic algorithms for treatment, whilst successful in many cases, can encourage over-treatment and expose patients unnecessarily to antibiotics [12]. Improved diagnosis relying on part technology and part syndromic management can reduce uncertainty about whether to treat with antibiotics or not.

Consistent with the universal health coverage strategic direction of improving efficiency in service delivery through improved technology, taken to scale, the advent of simple diagnostic tests could help reduce the need for mass administration of antibiotics and enable more precise prescription in many cases. The rapid and accurate establishment of a microbial cause is fundamental to quality care. New tests are needed that can identify a specific pathogen or at a minimum, distinguish between bacterial and viral infections, and also provide information on susceptibility to antimicrobial agents. There is no consensus on the type of diagnostics research and development should aim for [41]. When new diagnostic tests are developed, clinical factors and investigations on effectiveness in terms of antibiotic resistance, antibiotics use, or patient outcomes are less emphasized in their assessment in favour of efficacy considerations. The local context matters; there is also lack of clarity about how LMICs might take up these new diagnostic tests, in the context of different speed, robustness of system, cost, or user-friendliness [42]. In the meantime, existing, simple tests are still not widely used. However, there are significant challenges to the development, regulatory approval, and clinical integration of diagnostic tests that use these new technologies [43]. There is a key opportunity for private, non-profit and academic institutions to collaborate on this issue.

#### Affordability

A key element in getting access to care to the community will be the affordability of the antimicrobials [44]. Principles of both stewardship and global solidarity suggest that pricing and financing of novel antimicrobials at the national and subnational level must be undertaken with an eye towards innovative mechanisms [45]. There is a need to reduce the financial burden of health care in general on poor families, as emphasised in universal health coverage strategies. When considering the national schemes of government provided health insurance, measures to reduce the cost of antimicrobials to patients need to be complemented by actions to ensure these drugs are used appropriately, therefore coupled with treatment guidelines, effective monitoring and surveillance and alleviating perverse incentives.

In many pluralistic health systems the reliance on informal providers for antibiotics will mean the provision of universal health insurance will take time to be effective, therefore other measures will have to temporarily ensure access to effective treatments is increased. One option is for government, donor agencies and/or philanthropic organisations to reduce the cost of antimicrobials through more effective procurement from manufacturers and/or supplying drugs at a subsidised price [46].

With funds being allocated to the research and development of new antibiotic therapies in high-income countries we are likely to also see the limitation of new therapies to preserve their efficacy. Therefore a matching of funds needs to be prioritised for measures to increase appropriate access to common treatments globally. Such as, funding measures to reduce financial barriers to access, but also reducing exposure to infection and susceptibility to infections. This type of combined investment strategy is essential to garner wide political support, otherwise preventing the production and commercial use of any new therapies will be difficult [47].

#### Governance

The implementation of a sustained effort to achieve system-wide changes in the use both of existing, effective antibiotics and future, new antibiotics requires informed and committed collaboration at national and global levels [48]. In May 2015 the WHO released a global action plan on antibiotic resistance, but it remains to be seen whether effective global governance institutions can be created. There are numerous initiatives currently being conducted by various stakeholders and from different perspectives. Currently, there is no analysis as to how the host of initiatives function cohesively at the global level. New coordination and financing mechanisms, some of which must be organized globally utilizing global governance instruments and mechanisms are essential to tackling AMR. Although tracking progress on antibiotic resistance containment must be a prerogative of each national government, it is vital to develop monitoring and evaluation frameworks that allow for information sharing between countries regionally and globally.

Effective governance of antibiotics is key to the sustainable access and use challenge, this will involve effective regulation, the involvement of all actors and effective markets. National level political commitments, frameworks and institutions are also important [21]. For example, the creation of a high-level task force to oversee national efforts contributed to Sweden’s success in limiting antibiotic use [49]. Countries that have implemented comprehensive national strategies have been the most successful in controlling resistance [50]. Additionally, countries with cases of antibiotic resistance have found a targeted national approach successful eg, the UK for control of MRSA and Clostridium difficile [51] and the USA has implemented various initiatives [52]. However, these programmes need time and patience to be set up and need to be backed by visionary governments with adequate funding. A stepwise approach to a national strategy according to a contextualised and prioritised road map might be the best way forward for most settings. In resource-poor countries, there has been much less progress, although China, Vietnam and India notably have made important steps recently [53].

Barriers to the implementation of effective and sustainable programmes exist in many regions of the world. The bottlenecks for implementing stewardship in both resource rich and poor countries are often strikingly similar, largely as a result of insufficient leadership, commitment, and funding [54]. One major challenge in countries with weak management and governance structures is to involve powerful organisations in partnership arrangements, while protecting the interests of the relatively poor and powerless [48]. For example, pharmaceutical companies could make a substantial contribution towards improving antibiotic use but there is a tension between their search for short-term profits and the longer-term benefits of ensuring they are only used when needed. This raises questions about the degree to which large companies can be made accountable to local stakeholders and the potential role of global agreements on standards of behaviour. The increasing global presence of companies from rapidly growing middle-income countries and the consequent involvement of their governments in governance arrangements is creating another level of complexity, since these important global actors are concurrently building institutions to make their own pluralistic health systems more coherent. Governments have a key leadership role in overseeing the creation and oversight of these institutions. This may work better if other strong actors, who can express the interests of the different stakeholders, are involved. These could be strong NGOs, citizen organisations, faith based organisations, professional associations and so forth. There is limited evidence about the approaches that work well in building institutions in low and middle-income countries [55], however, exploring the possibilities of innovative partnerships will be key in tackling AMR. Acknowledging the presence of informal providers of drugs and services in LMICs and developing ways of creating “safer informality” will be essential in creating truly effective and representative partnerships. Key to governance will be consensus and coalition building amongst stakeholders; building shared visions of just and sustainable use and understanding and appeasing areas of competing interest.

### Sustainable future solutions in a complex world

Effective interventions will need to consist of a package of components – one approach is unlikely to suit all settings [56]. Based on the potential areas of intervention discussed above a number of key challenges and areas for further research present themselves. Table 1 provides an overview of the themes, rationale and variables that need to be explored in order to develop sustainable future access and appropriate use interventions for antibiotics. A number of possible interventions have an apparent simplicity, but in fact, they influence complex medical and evolutionary landscapes, that is they might result in many other effects, variable in different places and some of them eventually unwanted. Apparently ‘simple’ interventions are frequently complex and unpredictable in their effects [57]. Complexity influences interventions not only because of the number of interacting components, but also because of the number and difficulty of behaviours required by those delivering or receiving the intervention [58]; the number of groups or organizational levels targeted by the intervention [59], the number and variability of outcomes; and the degree of flexibility or tailoring of the intervention permitted [60]. A combination of synergistic interventions tailored to the wider ecological context and specific circumstances with the requisite monitoring of outcomes is likely the most efficient approach.

**Table 1:** Variables to explore for sustainable future access and appropriate use interventions

Theme	Rationale	Variables to explore for intervention
Access strategies	How should antibiotics be made available to all members of a community?	Roll out options, referral patterns, training community health workers to prescribe appropriately, exclusive vendor availability
Antibiotic quality	Measures to ensure antibiotic quality	Roles of different actors, effective technologies for low resource settings, drivers of quality
Decision making and help seeking (unlocking capabilities)	Strategies to enable people to treat infections when necessary while reducing risks of resistance	Suppliers of advice and drugs, role of financial incentives, assessment of risk and need, professional and social norms, understandings of disease and antibiotics, ideas of entitlement, design of packaging
Therapeutic and dosing strategies	Optimising drug use strategies based on the scientific, economic, social and epidemiological context	Explore antibiotic combinations, co-administration, co-formulation, cycling, best practice for frequency and adherence to dosing strategies
Use of diagnostics	How can diagnostics improve diagnosis and treatment and be relevant in low resource settings	Dual diagnosis of infection and resistance in low resource settings, meeting the needs of populations, integration with surveillance, effects on access to care, treatment-seeking behaviour or supply stock-outs, prescription/antibiotic use
Exploring integration of new strategies	Transmission of health behaviour messaging integration of appropriate use measures into everyday practices. Explore innovative ways of tracking, diagnosis, treatment, reporting, messaging and the surveillance of resistance and antibiotic use	How can mobile health technology be incorporated to improve diagnosis, treatment and surveillance; can social media be used to encourage appropriate use of new/existing therapies; role of pharmaceutical companies and appropriate use
The role of markets and market actors	Effective strategies for involving players at every level in the market (local, national, regional, international) and aligning incentives	Roles/responsibilities for information transmission, guideline adherence, positive incentive creation, measures to improve access and reduce resistance
Consensus and coalition building	Building (and negotiating) shared visions of just and sustainable use	Mapping competing understandings and interests of relevant organisations and associations; Building of coalitions for change
Governance	Effective mechanisms at the community and regional level for ensuring sustainable access and use of antibiotics	Agreed roles and responsibilities, effective funding streams, harmonisation where possible
Evaluation of systems	Observe impact of interventions	Other health consequences, clinical outcomes of AMR, resistance in the environment, health seeking behaviour and wider social consequences (economic, networks)
	Identify unintended consequences	

The idea of antibiotic mainstreaming, i.e. - always to consider the effects of various types of interventions and various types of decisions on future availability of antibiotics, could be a means to raise awareness and integrate potential behaviour modification with respect to antibiotics in all aspects of society [21].

## Conclusions

Antibiotics are different from all other medicines in that the effects of their use extend beyond individual patients. The societal effects of antibiotic use justifies that measures need to ensure they should be accessed, prescribed, dispensed and used appropriately and accurately based on robust scientific evidence. In order to create sustainable future solutions for just access and appropriate use of antibiotics interventions need to reflect the complex adaptive system of antibiotic use and availability. When considering this multi-tiered system, set within broader epidemiological and ecological contexts, the potential areas for synergy and conversely the potential unintended consequences need to be considered. With deeper understanding a number of key themes present opportunities for intervention: access strategies, measures to ensure the quality of antibiotics, measures to encourage just and sustainable decision making and help seeking, effective therapeutic and dosing strategies and the use of accurate diagnostics. The achievement of progress towards adopting and integrating interventions hinges on effective partnerships and coalition building, accurate evaluation systems and effective governance mechanisms. The activities of a large number of stakeholders will need to be aligned. This will involve new kinds of partnerships, a deliberative process bringing in different voices reflecting the complexity of the issue. Such partnerships will require a balance of interests and belief that the benefits of partnership outweigh any losses. Partnerships will need to exist at regional, national and global levels and involve public, private, formal and informal sectors and will likely go beyond traditional health system boundaries. In many settings this will present challenges and feasibility will vary in different settings. There are indeed many gaps and challenges in the current understanding of antibiotic access and use, but identifying the levers for change as part of a wider system clearly identifies areas for immediate action and ways to engender a long-term sustained change for antibiotic access and use.

## Abbreviations

AMR: Antimicrobial resistance
LMIC: Low and middle income country
MRSA: Methicillin Resistant Staphylococcus Aureus
NGO: Non-Governmental Organization
WHO: World Health Organization

## References

[1] Maddocks S (2013). Antimicrobial resistance: global problems need global solutions. Med J Aust.

[2] Morgan DJ, Okeke IN, Laxminarayan R, Perencevich EN, Weisenberg S (2011). Non-prescription antimicrobial use worldwide: a systematic review. Lancet Infect Dis.

[3] Laxminarayan R, Duse A, Wattal C, Zaidi AKM, Wertheim HFL, Sumpradit N, Vlieghe E, Hara GL, Gould IM, Goossens H (2013). Antibiotic resistance? the need for global solutions. Lancet Infect Dis.

[4] Peters DH, Bloom G (2013). Health care: order health systems in developing world. Nature.

[5] Tomson G, Vlad I (2014). The need to look at antibiotic resistance from a health systems perspective. Ups J Med Sci.

[6] Paina L, Bennett S, Ssengooba F, Peters DH (2014). Advancing the application of systems thinking in health: exploring dual practice and its management in Kampala, Uganda. Health Res Policy Syst/BioMed Central.

[7] Bloom G, Standing H, Lloyd R (2008). Markets, information asymmetry and health care: towards new social contracts. Soc Sci Med.

[8] Chandy SJ, Mathai E, Thomas K, Faruqui AR, Holloway K, Lundborg CS (2013). Antibiotic use and resistance: perceptions and ethical challenges among doctors, pharmacists and the public in Vellore, South India. Indian J Med Ethics.

[9] Sudhinaraset M, Ingram M, Lofthouse HK, Montagu D (2013). What is the role of informal healthcare providers in developing countries? A systematic review. PLoS One.

[10] Van der Geest S, Whyte SR, Hardon A (1996). The anthropology of pharmaceuticals: a biographical approach.

[11] Radyowijati A, Haak H (2003). Improving antibiotic use in low-income countries: an overview of evidence on determinants. Soc Sci Med.

[12] Nigel J Garrett, Nuala McGrath, Adrian MindelSex Transm Infect sextrans-2016-052581. 2016. doi:10.1136/sextrans-2016-052581.

[13] Reed EE, Stevenson KB, West JE, Bauer KA, Goff DA (2013). Impact of formulary restriction with prior authorization by an antimicrobial stewardship program. Virulence.

[14] van Limburg M, Sinha B, Lo-Ten-Foe JR, van Gemert-Pijnen JE (2014). Evaluation of early implementations of antibiotic stewardship program initiatives in nine Dutch hospitals. Antimicrob Resist Infect Control.

[15] Heyman G, Cars O, Bejarano M-T, Peterson S (2014). Access, excess, and ethics—towards a sustainable distribution model for antibiotics. Ups J Med Sci.

[16] Toutain PL, Bousquet-Melou A (2013). The consequences of generic marketing on antibiotic consumption and the spread of microbial resistance: the need for new antibiotics. J Vet Pharmacol Ther.

[17] Nayyar GM, Breman JG, Newton PN, Herrington J (2012). Poor-quality antimalarial drugs in southeast Asia and sub-Saharan Africa. Lancet Infect Dis.

[18] Newton PN, Green MD, Fernandez FM (2010). Impact of poor-quality medicines in the ‘developing’ world. Trends Pharmacol Sci.

[19] Tabernero P, Fernandez FM, Green M, Guerin PJ, Newton PN (2014). Mind the gaps--the epidemiology of poor-quality anti-malarials in the malarious world--analysis of the WorldWide Antimalarial Resistance Network database. Malar J.

[20] Attaran A, Barry D, Basheer S, Bate R, Benton D, Chauvin J, Garrett L, Kickbusch I, Kohler JC, Midha K (2012). How to achieve international action on falsified and substandard medicines. BMJ.

[21] Stalsby Lundborg C, Tamhankar AJ (2014). Understanding and changing human behaviour--antibiotic mainstreaming as an approach to facilitate modification of provider and consumer behaviour. Ups J Med Sci.

[22] Hawkings NJ, Butler CC, Wood F (2008). Antibiotics in the community: a typology of user behaviours. Patient Educ Couns.

[23] Planta MB (2007). The role of poverty in antimicrobial resistance. J Am Board Fam Med.

[24] Zarb P, Goossens H (2012). Human use of antimicrobial agents. Rev Sci Tech.

[25] Roque F, Herdeiro MT, Soares S, Teixeira Rodrigues A, Breitenfeld L, Figueiras A (2014). Educational interventions to improve prescription and dispensing of antibiotics: a systematic review. BMC Public Health.

[26] Bediang G, Stoll B, Elia N, Abena JL, Nolna D, Chastonay P, Geissbuhler A (2014). SMS reminders to improve the tuberculosis cure rate in developing countries (TB-SMS Cameroon): a protocol of a randomised control study. Trials.

[27] Sumpradit N, Chongtrakul P, Anuwong K, Pumtong S, Kongsomboon K, Butdeemee P, Khonglormyati J, Chomyong S, Tongyoung P, Losiriwat S (2012). Antibiotics Smart Use: a workable model for promoting the rational use of medicines in Thailand. Bull World Health Organ.

[28] MacDougall C, Polk RE (2005). Antimicrobial stewardship programs in health care systems. Clin Microbiol Rev.

[29] Masiero G, Filippini M, Ferech M, Goossens H (2010). Socioeconomic determinants of outpatient antibiotic use in Europe. Int J Public Health.

[30] Mintzes B, Barer M, Lexchin J, Bassett KL (2005). Introduction of direct-to-consumer advertising of prescription drugs in Canada: an opinion survey on regulatory policy. Res Soc Adm Pharm.

[31] Davey P, Brown ED, Charani E (2013). Interventions to improve antibioitc prescribing practices for hospital inpatients. Cochrane Database Syst Rev.

[32] McGuire TM, Smith J, Del Mar C (2015). The match between common antibiotics packaging and guidelines for their use in Australia. Aust N Z J Public Health.

[33] Bonhoeffer S, Lipsitch M, Levin BR (1997). Evaluating treatment protocols to prevent antibiotic resistance. Proc Natl Acad Sci U S A.

[34] Gjini E, Brito PH (2016). Integrating antimicrobial therapy with host immunity to fight drug-resistant infections: classical vs. adaptive treatment. PLoS Comput Biol.

[35] Day T, Read AF. Does High-Dose Antimicrobial Chemotherapy Prevent the Evolution of Resistance? PLoS Comput Biol. 2016;12(1):1-20. 10.1371/journal.pcbi.1004689 PMC4731197 26820986

[36] Baquero F, Coque TM, Canton R (2014). Counteracting antibiotic resistance: breaking barriers among antibacterial strategies. Expert Opin Ther Targets.

[37] Worthington RJ, Melander C (2013). Combination approaches to combat multidrug-resistant bacteria. Trends Biotechnol.

[38] Worthington RJ, Melander C (2013). Overcoming resistance to beta-lactam antibiotics. J Org Chem.

[39] Gelband H, Laxminarayan R (2015). Tackling antimicrobial resistance at global and local scales. Trends Microbiol.

[40] Laxminarayan R (2012). Economics of antibiotic resistance: a matter of life and death. Milken Rev.

[41] Kessel M (2015). Why microbial diagnostics need more than money. Nat Biotechnol.

[42] Okeke IN, Sosa A. Antibiotic resistance in Africa—discerning the enemy and plotting a defence. Alliance for the Prudent Use of Antibiotics (APUA).http://www.tufts.edu/med/apua/about_issue/africahealth.pdf .[Accessed January 2016].

[43] Caliendo AM, Gilbert DN, Ginocchio CC, Hanson KE, May L, Quinn TC, Tenover FC, Alland D, Blaschke AJ, Bonomo RA (2013). Better tests, better care: improved diagnostics for infectious diseases. Clin Infect Dis.

[44] Kesselheim AS, Outterson K (2010). Fighting antibiotic resistance: marrying new financial incentives to meeting public health goals. Health Aff (Millwood).

[45] Kesselheim AS, Outterson K (2011). Improving antibiotic markets for long-term sustainability. Yale J Health Policy Law Ethics.

[46] Outterson K, McDonnell A (2016). Funding antibiotic innovation with vouchers: recommendations on how to strengthen a flawed incentive policy. Health Aff (Millwood).

[47] So AD, Gupta N, Brahmachari SK, Chopra I, Munos B, Nathan C, Outterson K, Paccaud JP, Payne DJ, Peeling RW (2011). Towards new business models for R&D for novel antibiotics. Drug Resist Updat.

[48] Collignon P, Athukorala P-c, Senanayake S, Khan F (2015). Antimicrobial resistance: the major contribution of poor governance and corruption to this growing problem. PLoS One.

[49] Molstad S, Cars O, Struwe J (2008). Strama--a Swedish working model for containment of antibiotic resistance. Euro Surveill.

[50] Leung E, Weil DE, Raviglione M, Nakatani H, on behalf of the World Health Organization World Health Day Antimicrobial Resistance Technical Working G (2011). The WHO policy package to combat antimicrobial resistance. Bull World Health Organ.

[51] Stone SP, Fuller C, Savage J, Cookson B, Hayward A, Cooper B, Duckworth G, Michie S, Murray M, Jeanes A (2012). Evaluation of the national Cleanyourhands campaign to reduce Staphylococcus aureus bacteraemia and Clostridium difficile infection in hospitals in England and Wales by improved hand hygiene: four year, prospective, ecological, interrupted time series study. BMJ.

[52] Schwaber MJ, Lev B, Israeli A, Solter E, Smollan G, Rubinovitch B, Shalit I, Carmeli Y, Israel Carbapenem-Resistant Enterobacteriaceae Working G (2011). Containment of a country-wide outbreak of carbapenem-resistant Klebsiella pneumoniae in Israeli hospitals via a nationally implemented intervention. Clin Infect Dis.

[53] Wertheim HF, Chandna A, Vu PD, Pham CV, Nguyen PD, Lam YM, Nguyen CV, Larsson M, Rydell U, Nilsson LE (2013). Providing impetus, tools, and guidance to strengthen national capacity for antimicrobial stewardship in Viet Nam. PLoS Med.

[54] Daulaire N, Bang A, Tomson G, Kalyango JN, Cars O (2015). Universal access to effective antibiotics is essential for tackling antibiotic resistance. J Law Med Ethics.

[55] Fukuyama F (2004). The imperative of state building. J Democr.

[56] Baquero F, Lanza VF, Cantón R, Coque TM (2015). Public health evolutionary biology of antimicrobial resistance: priorities for intervention. Evol Appl.

[57] Read AF, Day T, Huijben S (2011). The evolution of drug resistance and the curious orthodoxy of aggressive chemotherapy. Proc Natl Acad Sci U S A.

[58] Charani E, Castro-Sanchez E, Holmes A (2014). The role of behavior change in antimicrobial stewardship. Infect Dis Clin North Am.

[59] Grundmann H (2014). Towards a global antibiotic resistance surveillance system: a primer for a roadmap. Ups J Med Sci.

[60] Viana LV, Gross JL, Azevedo MJ (2014). Dietary intervention in patients with gestational diabetes mellitus: a systematic review and meta-analysis of randomized clinical trials on maternal and newborn outcomes. Diabetes Care.

